# Humoral immune response to HTLV-1 basic leucine zipper factor (HBZ) in HTLV-1-infected individuals

**DOI:** 10.1186/1742-4690-10-19

**Published:** 2013-02-13

**Authors:** Yoshimi Enose-Akahata, Anna Abrams, Raya Massoud, Izabela Bialuk, Kory R Johnson, Patrick L Green, Elizabeth M Maloney, Steven Jacobson

**Affiliations:** 1 Viral Immunology Section, Neuroimmunology Branch, National Institute of Neurological Disorders and Stroke, National Institutes of Health, Bethesda, MD, USA; 2 Department of General and Experimental Pathology, Medical University of Bialystok, Bialystok, Poland; 3 Bioinformatics Section, Division of Intramural Research, National Institute of Neurological Disorders and Stroke, National Institutes of Health, Bethesda, MD, USA; 4 Center for Retrovirus Research, The Ohio State University, Columbus, OH, USA; 5 Formerly of the Viral Epidemiology Branch, Division of Cancer Epidemiology and Genetics, National Cancer Institute, National Institutes of Health, Bethesda, MD, USA; 6 Current affiliation: Center for Drug Evaluation and Research, Office of Surveillance and Epidemiology, Food and Drug Administration, Silver Spring, MD, USA

**Keywords:** HTLV-1, Antibody, HAM/TSP, ATL, Asymptomatic carriers, Serum, CSF

## Abstract

**Background:**

Human T cell lymphotropic virus type 1 (HTLV-1) infection can lead to development of adult T cell leukemia/lymphoma (ATL) or HTLV-1-associated myelopathy/tropical spastic paraparesis (HAM/TSP) in a subset of infected subjects. HTLV-1 basic leucine zipper factor (HBZ) gene has a critical role in HTLV-1 infectivity and the development of ATL and HAM/TSP. However, little is known about the immune response against HBZ in HTLV-1-infected individuals. In this study, we examined antibody responses against HBZ in serum/plasma samples from 436 subjects including HTLV-1 seronegative donors, asymptomatic carriers (AC), ATL, and HAM/TSP patients using the luciferase immunoprecipitation system.

**Results:**

Immunoreactivity against HBZ was detected in subsets of all HTLV-1-infected individuals but the test did not discriminate between AC, ATL and HAM/TSP. However, the frequency of detection of HBZ-specific antibodies in the serum of ATL patients with the chronic subtype was higher than in ATL patients with the lymphomatous subtype. Antibody responses against HBZ were also detected in cerebrospinal fluid of HAM/TSP patients with anti-HBZ in serum. Antibody responses against HBZ did not correlate with proviral load and HBZ mRNA expression in HAM/TSP patients, but the presence of an HBZ-specific response was associated with reduced CD4^+^ T cell activation in HAM/TSP patients. Moreover, HBZ-specific antibody inhibited lymphoproliferation in the PBMC of HAM/TSP patients.

**Conclusions:**

This is the first report demonstrating humoral immune response against HBZ associated with HTLV-I infection. Thus, a humoral immune response against HBZ might play a role in HTLV-1 infection.

## Background

Human T cell lymphotropic virus 1 (HTLV-1) infects 20 million people worldwide [1]. While the majority of infected individuals are asymptomatic carriers (AC) of the virus, 5-10% of infected people develop either adult T cell leukemia/lymphoma (ATL) [2] or a chronic, progressive, neurological disease termed HTLV-1-associated myelopathy/tropical spastic paraparesis (HAM/TSP) [3,4]. HAM/TSP is characterized by perivascular inflammatory infiltrates in the brain and spinal cord, with a predominance of HTLV-1-specific CD8^+^ T cells [5,6]. High frequencies of these effector cells have been demonstrated in peripheral blood with even higher frequencies in cerebrospinal fluid (CSF) of patients with HAM/TSP [7-9], and robust humoral responses against HTLV-1 antigens that can be detected in the CSF as well as the serum [4,10]. While the cellular and humoral immune responses against HTLV-1 play crucial, protective roles in HTLV-1 infection, chronically activated immune responses have been suggested to underlie the pathogenesis of HAM/TSP [11]. Therefore, characterization of HTLV-1-specific immune responses may provide evidence of immune dysregulation during disease progression in HAM/TSP patients, and may help identify novel immunotherapeutic targets in HTLV-1-related diseases.

Despite strong HTLV-1-specific immune responses, HTLV-1 proviral loads are significantly elevated in HAM/TSP patients compared to AC [12]. Increased expression particularly of the trans-activating viral gene encoding HTLV-1 Tax induces the expression of various cellular genes, including IL-2, IL-15, and their receptors [13-16], which directly contributes to lymphocyte activation in HAM/TSP patients [9,17]. A novel gene, HTLV-1 basic leucine zipper factor (HBZ), encoded by the minus strand of the HTLV-1 proviral genome has been identified [18]. HBZ mRNA is ubiquitously expressed in all ATL cells and promotes the growth and survival of the leukemic cells [19]. HBZ protein was found to inhibit Tax-mediated viral gene transcription from the 5’ LTR and to selectively suppress the classical NF-κB pathway [18,20-23]. Previous *in vivo* studies also demonstrated that HBZ expression enhanced HTLV-1 infectivity, T cell proliferation and lymphoma [24-26]. Furthermore, HBZ mRNA expression was detected in HAM/TSP patients, and was correlated with proviral load and disease severity [27]. Since these findings suggested that HBZ has a critical role in HTLV-1 persistence and the development of ATL and HAM/TSP, it is important to define HBZ-specific immune responses in HTLV-1-infected individuals.

Recent evidence has shown that HBZ is an immunogenic protein recognized by HBZ-specific CTL clones [28,29]. HBZ-specific CD8^+^ T cells are detected in AC and HAM/TSP patients, and HBZ-specific CTL clones were able to lyse naturally infected cells isolated from AC and HAM/TSP patients, but not ATL patients [28,29]. Despite recent studies on HBZ-specific cellular immune responses, there are no reports on the humoral immune responses to HBZ. We recently reported that a luciferase immunoprecipitation system (LIPS), a highly sensitive, quantitative technology, could efficiently detect HTLV-1 antigen-specific antibody responses in serum of HTLV-1-infected individuals [30,31]. Since the LIPS assay can detect antibody responses against multiple antigens, profiling of HTLV-1-specific antibody responses using LIPS demonstrated a differential pattern of antibody responses for HTLV-1 Gag, Env and Tax between HTLV-1-infected and uninfected subjects as well as between the AC and ATL and HAM/TSP patients [30,31]. Here we optimized the LIPS assay for detection of immunoreactivity against HBZ, and first determined antibody responses against HBZ in HTLV-1-infected individuals.

## Results

### Characteristics of the study population

The demographic characteristics of the study groups are summarized in Table 1. Among Jamaican subjects, the mean ages of the study groups varied from 38 years of age in the HTLV-1-seronegative donor (ND) group to 47 years in the HAM/TSP group (p = 0.0003). The majority of each group was comprised of females, although the proportion of females in each group ranged from 53.9% in the ATL group to 83.5% in the AC group (p < 0.0001). All the study groups were predominantly of African-descent. Among the NIH subjects, the mean ages of the study groups varied from 45 years in the ND group to 58 years in the AC group (p = 0.0052). The proportion of females in each group ranged from 24.0% in the ND group to 75.0% in the AC group (p = 0.0074). The proportion of African-descent and Caucasian-descent were equal in the ND and the AC group; there were slightly more individuals of African-descent in the HAM/TSP group.

**Table 1:** Distribution of demographic factors among study groups

**Jamaican cohort**				
**Factor**	**ND (n = 73)**	**AC (n = 133)**	**ATL (n = 89)**	**HAM/TSP (n = 49)**
Gender (n[%])				
Male	15 [20.5]	22 [16.5]	41 [46.1]	13 [26.5]
Female	58 [79.5]	111 [83.5]	48 [53.9]	36 [73.5]
Race (n[%])				
African-descent	72 [98.6]	132 [99.2]	86 [96.6]	42 [85.7]
Caucasian-descent	0 [0.0]	0 [0.0]	0 [0.0]	1 [2.0]
Other	0 [0.0]	1 [3.0]	0 [0.0]	2 [4.1]
Missing	1 [1.4]		3 [3.4]	4 [8.2]
Age†				
Mean ± s.e.m.	38.5 ± 1.5	42.2 ± 1.2	45.5 ± 1.7	47.4 ± 1.8
Range	(18 – 76)	(18 – 75)	(18 – 80)	(14 – 74)
**NIH cohort**				
**Factor**	**ND (n = 25)**	**AC (n = 12)**	**HAM/TSP (n = 55)**	
Gender (n[%])				
Male	19 [76.0]	3 [25.0]	17 [30.9]	
Female	11 [24.0]	9 [75.0]	38 [69.1]	
Race (n[%])				
African-descent	11 [44.0]	5 [41.7]	36 [65.5]	
Caucasian-descent	11 [44.0]	5 [41.7]	14 [25.5]	
Other	3 [12.0]	2 [16.6]	5 [9.1]	
Age				
Mean ± s.e.m.	45.6 ± 2.4	58.9 ± 3.6	51.75 ± 1.5	
Range	(24 – 73)	(36 – 75)	(25 – 75)	

†Information on age was missing for 1 HAM/TSP subject from Jamaican cohort.

### Antibody responses against HBZ in serum/plasma

**Figure 1:**
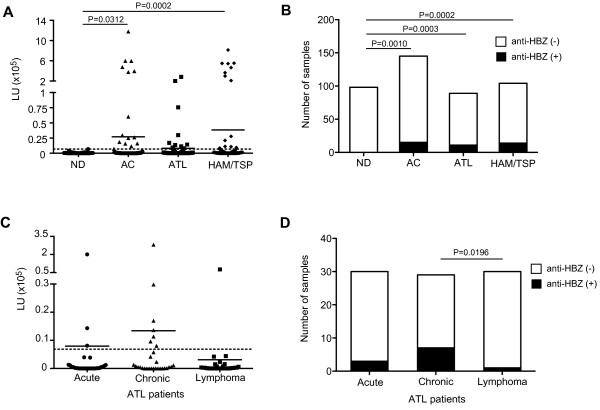
**Antibody responses against HBZ from serum/plasma of ND, AC, ATL patients and HAM/TSP patients. **(**A**) Comparison of antibody responses against HBZ in serum/plasma of ND, AC, ATL and HAM/TSP patients using Mann–Whitney Test. The data were obtained from 98 ND, 145 AC, 89 ATL patients and 104 HAM/TSP patients. Antibody responses against HBZ were detected in HTLV-1-infected groups (AC, ATL and HAM/TSP). The horizontal line represents the mean. Dotted line represents cut-off value (6853LU). (**B**) Frequency of subjects with antibody response against HBZ in serum/plasma of ND, AC, ATL and HAM/TSP patients. The distribution of subjects with antibody response against HBZ among the groups was compared by Chi-Square Test. (**C**) Comparison of antibody responses against HBZ in serum of ATL patients by ATL subtypes using Mann–Whitney Test. The data were obtained from 30 acute, 29 chronic and 30 lymphoma ATL patients. The horizontal line represents the mean. Dotted line represents cut-off value (6853LU). (**D**) Frequency of subjects with antibody response against HBZ in serum of ATL patients. The distribution of subjects with antibody response against HBZ among the groups was compared by Chi-Square Test.

Antibody responses for HBZ were analyzed in the separate groups of Jamaican and NIH subjects. There were no significant differences in frequency or magnitude of anti-HBZ antibody responses in serum/plasma between Jamaican and NIH subjects (data not shown) so they were combined in the remaining analyses, yielding a total of 436 serum/plasma samples obtained from ND, AC, ATL patients and HAM/TSP patients. Strong mean antibody levels against HBZ were detected in the HTLV-1-infected groups, including AC, ATL patients and HAM/TSP patients, compared to the ND group (Figure 1A and Table 2). Although the mean antibody level against HBZ among the ATL subjects did not differ significantly from those of the ND group, the differences of the mean antibody level were statistically significant for the AC and HAM/TSP groups relative to the ND group (Figure 1A). Within each HTLV-1-infected group, robust anti-HBZ antibody responses (209751.9-1180625.0 LU) were observed in subsets of individuals, but among the HTLV-1-infected groups, the mean anti-HBZ antibody levels were not significantly different (Figure 1A). When the data were analyzed as the percent of positive responders above a negative threshold (6853 LU; dotted line in Figure 1A), immunoreactivity against HBZ was detected in 10.34% (15/145) of AC, 12.36% (11/89) of ATL patients, and 13.46% (14/104) of HAM/TSP patients (Figure 1B and Table 2). The frequency of immunoreactivity in each of the HTLV-1-infected groups was significantly higher than the ND group, but again there were no statistically significant differences between the HTLV-1-infected groups (Figure 1B). In addition, the Four-Way Analysis of Variance (ANOVA) model with interactions using race, gender, age and study group as factors showed no significant differences of the level of immunoreactivity against HBZ by gender, race and age between each study group (data not shown). There were also no significant differences in the frequency of immunoreactivity against HBZ by gender, race and age between each study group (data not shown).

**Table 2:** Antibody responses against HTLV-1 HBZ

**All subjects^**				
**HTLV-1 Antibody**	**ND (n = 98)**	**AC (n = 145)**	**ATL (n = 89)**	**HAM/TSP (n = 104)**
Anti-HBZ				
Mean	626.5	27102	8112	38413
Median	99.0	375.9	256.6	692.2
Range	(0–6853)	(0–1180625)	(0–281419)	(0–815217)
St. dev.	1048	132838	37168	135893
				
Number of anti-	0	15	11	14
HBZ + subjects (%)*	(0)	(10.34)	(12.36)	(13.46)
**ATL subtype**				
**HTLV-1 Antibody**	**Acute (n = 30)**	**Chronic (n = 29)**	**Lymphoma (n = 30)**	
Anti-HBZ				
Mean	7967	13413	3132	
Median	165.1	535.0	166.0	
Range	(0–201062)	(0–281419)	(0–75697)	
St. dev.	36592	51994	13753	
			
Number of anti-	3	7	1	
HBZ + subjects (%)*	(10.0)	(24.14)	(3.33)	

^Data includes both Jamaican and NIH cohort.

* Cut off value: 6853LU (100% percentile of ND).

Since HBZ has been suggested to play a role in ATL, and ATL patients can be divided into the predominant clinically unique subtypes (acute, chronic or lymphoma), it was of interest to determine if serum anti-HBZ responses could discriminate among these subtypes. The mean anti-HBZ antibody levels were not significantly different among the ATL subtypes (Figure 1C). However, the prevalence of HBZ immunoreactivity was detected in 10.0% (3/30) of ATL patients with the acute subtype, 24.14% (7/29) of those with the chronic subtype, and 3.33% (1/30) of those with the lymphoma subtype, demonstrating that ATL patients with the chronic subtype showed significantly higher anti-HBZ prevalence compared to ATL patients with the lymphoma subtype (Figure 1D and Table 2). Collectively, these results demonstrated that 11.83% (40/338) of all HTLV-1-infected individuals, with or without HTLV-related diseases, had immunoreactivity against HTLV-1 HBZ.

### Antibody responses against HBZ did not correlate with those for Gag, Env and Tax, and HTLV-1 infection

**Figure 2:**
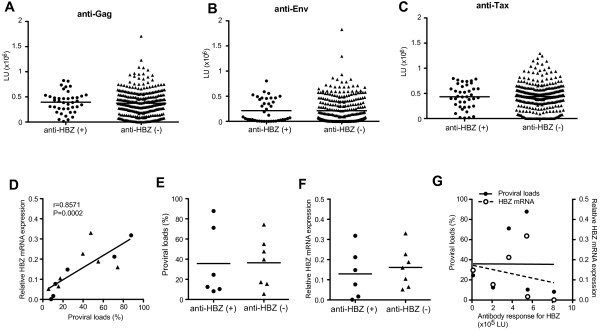
**Comparison of antibody responses against HTLV-1, proviral loads and HBZ mRNA expression of HTLV-1-infected individuals. **Comparison of antibody responses against Gag (**A**), Env (**B**) and Tax (**C**) in serum/plasma of HTLV-1-infected individuals with and without antibody response against HBZ by Mann–Whitney Test. The data were obtained from 338 HTLV-1-infected individuals; 145 AC, 89 ATL patients and 104 HAM/TSP patients. Anti-HBZ (−) group includes 130 AC, 78 ATL patients and 90 HAM/TSP patients. Anti-HBZ (+) group includes 15 AC, 11 ATL patients and 14 HAM/TSP patients. The horizontal line represents the mean. (**D**) Correlation of HTLV-1 proviral load with HBZ mRNA expression in 13 HAM/TSP patients including 6 patients with antibody response against HBZ (closed circles) and 7 patients without antibody response against HBZ (closed triangles) by Spearman’s correlation test. (**E**) Comparison of HTLV-1 proviral loads between HAM/TSP patients with and without antibody response against HBZ. HTLV-1 proviral loads were examined in PBMCs of HAM/TSP patients using Mann–Whitney Test. The horizontal line represents the mean. (**F**) Comparison of HBZ mRNA expression between HAM/TSP patients with and without antibody response against HBZ. The expression of HBZ mRNA was examined in PBMCs of HAM/TSP patients using Mann–Whitney Test. The horizontal line represents the mean. (**G**) Correlation of immunoreactivity against HBZ with HTLV-1 proviral loads (closed circles) and HBZ mRNA expression (opened circles) in 6 HAM/TSP patients with antibody response against HBZ by Spearman’s correlation test.

Since HBZ-specific immunoreactivity was detected in a subset of HTLV-1 infected subjects, we asked whether there is any relationship between HBZ-specific immunoreactivity and other immunological and virological markers of HTLV-1 infection, such as antibody responses to other HTLV-1 proteins and HTLV-1 viral gene expression. Antibody responses against Gag, Env or Tax did not differ significantly between HTLV-1-infected individuals with or without HBZ-specific immunoreactivity (Figure 2A-C). We also compared HTLV-1 proviral load and HBZ mRNA expression between HTLV-1-infected individuals with and without HBZ-specific immunoreactivity. Since PBMC were unavailable for the Jamaican subjects, cells were obtained from NIH HAM/TSP patients whose serum was tested for anti-HBZ antibody responses. These 13 HAM/TSP patients showed a range of HTLV-1 proviral loads in PBMCs between 5.6 and 87.8% (Figure 2D). Consistent with a previous report [27], HBZ mRNA was detectable in PBMCs of HAM/TSP patients and significantly correlated with proviral loads (Figure 2D). There was no significant difference of proviral loads between individuals with and without HBZ-specific immunoreactivity (Figure 2E); there was also no significant correlation between proviral load and the HBZ-specific antibody responses (Figure 2G). Moreover, the mean expression of HBZ mRNA from HAM/TSP patients’ PBMC was not associated with the detection of HBZ immunoreactivity (Figure 2F) or magnitude of serum anti-HBZ antibodies (Figure 2G). Thus, antibody responses for HBZ did not correlate with proviral loads or HBZ mRNA expression in HAM/TSP patients, consistent with previous studies that also failed to demonstrate a correlation between HBZ mRNA expression and HTLV-1 antibody titer in serum [27].

### Antibody responses against HBZ in CSF

**Figure 3:**
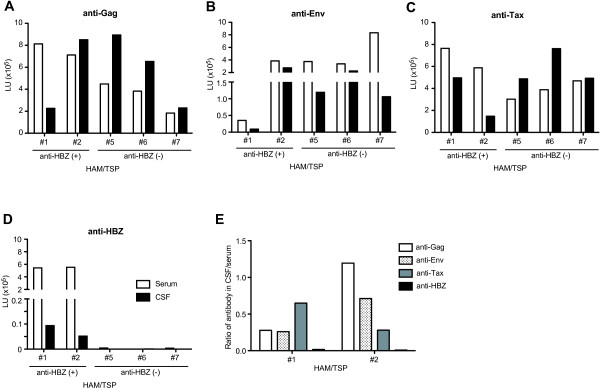
**Detection of antibody responses against HTLV-1 in serum and CSF of HAM/TSP patients. **The data were obtained from 5 HAM/TSP patients including 2 patients (#1 and #2) and 3 patients (#5, #6 and #7) with and without HBZ-specific antibody response, respectively. Antibody responses for Gag (**A**), Env (**B**), Tax (**C**) and HBZ (**D**) were examined in serum (opened bar) and in CSF (closed bar). (**E**) Ratio of immunoreactivities against HTLV-1 Gag, Env, Tax and HBZ in CSF to those in serum of two HAM/TSP patients with HBZ-specific antibody responses.

Since strong antibody responses against HTLV-1 antigens have been reported in both serum and CSF of HAM/TSP patients [10,32], we also examined antibody responses for HTLV-1 Gag, Env, Tax and HBZ in both serum and CSF samples of HAM/TSP patients with or without HBZ-specific antibody responses. Antibody responses for Gag, Env and Tax were detected in both serum and CSF samples of all five HAM/TSP patients (Figure 3A-C). Antibody responses for HBZ were only detected in CSF samples of HAM/TSP patients (#1 and #2) with HBZ-specific antibody responses in serum, but not in HAM/TSP patients (#5, #6 and #7) who were negative for HBZ-specific immunoreactivity in serum (Figure 3D). Moreover, HBZ-specific antibody responses in CSF were detected at lower levels compared to serum (Figure 3D). Comparison of antibody responses between CSF and serum revealed that the ratio of anti-HBZ antibody in CSF to serum was lower than the ratio of anti-Gag, anti-Env and anti-Tax in CSF to serum (Figure 3E).

### A role of HBZ-specific antibody in HTLV-1 infection

**Figure 4:**
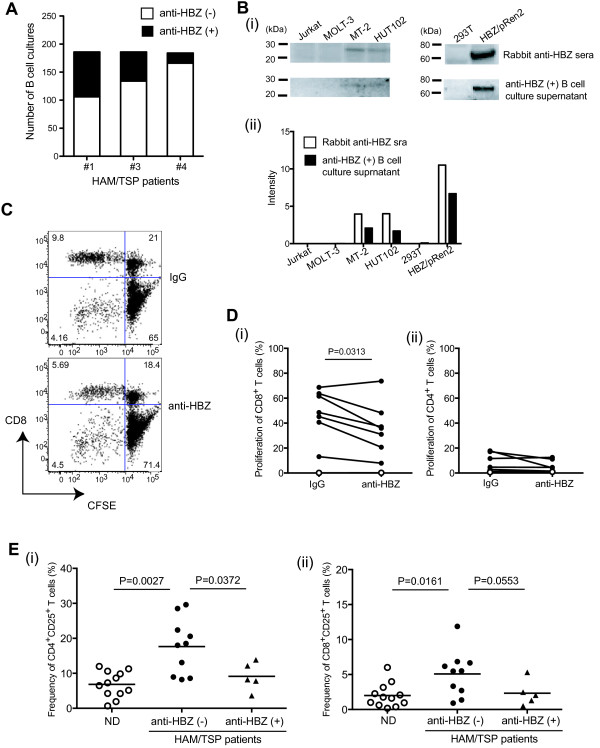
**Inhibitory effects of HBZ-specific antibody on T cell activation of HAM/TSP patients. **(**A**) Detection of immortalized memory B cells producing HBZ-specific antibodies in HAM/TSP patients. The immortalized B cells were generated from three HAM/TSP patients with anti-HBZ in serum, and production of HBZ-specific antibodies (closed bars) was detected using LIPS assay. (**B**) Detection of HBZ proteins using an anti-HBZ (+) B cell culture supernatant and rabbit anti-HBZ serum by western blot. The nuclear proteins were extracted from HTLV-1-uninfected (Jurkat and MOLT-3) and HTLV-I-infected (MT-2 and HUT102) cell lines and HBZ/pRen2-untransfected and transfected 293T cells. Detection of HBZ proteins was confirmed by western blot (i) representative image and (ii) the intensity of HBZ proteins. (**C**) Representative dot plots of CFSE staining in CD8^+ ^T cells of a HAM/TSP patient with HBZ-specific antibody or control IgG after culture for 6 days. (**D**) Inhibitory effects of HBZ-specific antibody on spontaneous proliferation of CD8^+ ^T cells (i) and CD4^+ ^T cells (ii) in PBMCs of HAM/TSP patients after culture for 6 days by Wilcoxson matched-pairs signed rank test. The data were obtained from 7 HAM/TSP patients without anti-HBZ response (closed circles) and one ND (opened circle) as control. (**E**) Comparison of frequencies of CD4^+^CD25^+ ^T cells (i) and CD8^+^CD25^+ ^T cells (ii) of NDs, HAM/TSP patients with and without antibody responses for HBZ by Mann–Whitney test. The data were obtained from twelve NDs, and five and ten HAM/TSP patients with and without antibody responses for HBZ, respectively. The horizontal line represents the mean.

Since HBZ is involved in both regulation of viral gene transcription and T-cell proliferation [18,20-23], we asked whether HBZ-specific antibody responses have potentially beneficial roles in suppressing immune activation in HAM/TSP patients. To confirm the inhibitory effect of HBZ-specific antibody on T cell activation, we further attempted to generate immortalized memory B cells producing HBZ-specific antibody from HAM/TSP patients and isolate the specific antibody from the B cell culture supernatants. Production of HBZ-specific antibodies was detected in 9.2-41.4% of immortalized memory B cell cultures from all three HAM/TSP patients with HBZ-specific antibody response (#1, #3 and #4; Figure 4A). As control, HTLV-I-specific antibodies to Gag, Env and Tax were also able to be detected in memory B cell pools of both HAM/TSP patients with and without HBZ-specific antibody response, but HBZ-specific antibody production was not detected in the memory B cell cultures of HAM/TSP patients without HBZ-specific antibody response (data not shown). To confirm the reactivity of HBZ-specific antibody produced from memory B cells, we examined HBZ protein detection using an anti-HBZ (+) B cell culture supernatant by western blot. The nuclear proteins were extracted from HTLV-1-uninfected (Jurkat and MOLT-3) and infected cell lines (MT-2 and HUT102) and HBZ/pRen2-untransfected and transfected 293 T cells. As shown in Figure 4 Bi, HBZ protein (25 kDa) and HBZ-*Renilla* luciferase (Ruc) fusion protein (61 kDa) was detected in nuclear protein extract of HTLV-1 infected cell lines (MT-2 and HUT102) and HBZ/pRen2 transfected 293T cells, respectively, using anti-HBZ (+) B cell culture supernatant. Rabbit anti-HBZ serum was used as a positive control and also reacted with the HBZ protein and HBZ-Ruc fusion protein similar to the anti-HBZ (+) B cell culture supernatant (Figure 4 Bi and ii). Furthermore, we isolated HBZ-specific IgG from supernatants of a memory B cell culture from HAM/TSP patient (#1), and examined the inhibitory effect on spontaneous lymphoproliferation in PBMCs of HAM/TSP patients without anti-HBZ response. The representative dot plots showed the inhibition of spontaneous proliferation by HBZ-specific IgG in PBMCs of a HAM/TSP patient without anti-HBZ response (Figure 4C). As shown in Figure 4 Di, HBZ-specific IgG significantly inhibited the spontaneous lymphoproliferation predominantly in CD8^+^ T cells of HAM/TSP patients without anti-HBZ response. Since CD4^+^ T cells of HAM/TSP patients showed significantly less proliferation than CD8^+^ T cells, it was difficult to observe an inhibitory effect of HBZ-specific IgG on CD4^+^ T cell proliferation in HAM/TSP patients without anti-HBZ response (Figure 4 Dii). Consistent with previous reports [33], the frequency of CD4^+^CD25^+^ T cells was higher in HAM/TSP patients without anti-HBZ antibody responses than those of ND, but HAM/TSP patients with anti-HBZ antibody responses showed significantly less CD4^+^CD25^+^ T cells compared to patients without anti-HBZ antibody responses (Figure 4 Ei). Also in CD8^+^ T cells, HAM/TSP patients without anti-HBZ antibody response showed significantly higher frequency of CD25^+^ cells than those of NDs (Figure 4 Eii). The frequency of CD8^+^CD25^+^ T cells was less in HAM/TSP patients with anti-HBZ response than those of patients without anti-HBZ response and approached significance (P = 0.0553; Figure 4 Eii). These results demonstrate that HBZ-specific antibody responses may have a role in suppressing T cell activation in HAM/TSP patients.

## Discussion

The HBZ gene is constitutively expressed in HTLV-1-infected cells, ATL cells and PBMC of HTLV-1-infected individuals [19,27,34] and is thought to be involved in both regulation of viral gene transcription and T-cell proliferation [18,20-23], suggesting that HBZ has a critical role in HTLV-1 infectivity and the development of HTLV-1-related diseases [24-27]. Therefore, it is important to define HBZ-specific immune responses in HTLV-1-infected individuals. In this study, we screened 436 serum/plasma samples from Jamaica and the United States, including NDs, ACs, ATL and HAM/TSP patients, and first defined the humoral immune response to HBZ in HTLV-1-infected individuals. The results demonstrated that antibody responses for HBZ were detected in 11.8% of HTLV-1-infected individuals. The frequency of antibody response for HBZ was low compared to a high frequency of antibody responses for Gag, Env and Tax (99.3%, 92.3% and 93.0%, respectively) as previously described [31]. Recently, it was reported that HBZ-specific CD8^+^ T cells were detected in HTLV-1-infected individuals, and HBZ-specific CTL clones were able to lyse naturally infected cells [28,29]. Likewise, it was demonstrated that the predicted binding affinity of HLA molecules to HBZ peptides is significantly weaker than that of Tax peptides and that the frequency of HBZ-specific CD8^+^ T cells is significantly lower than the frequency of Tax-specific CD8^+^ T cells [29]. Our results support previous reports [28,29] suggesting that HBZ is an immunogenic protein although HBZ-specific immune responses appear to be lower compared to the other HTLV-1 immunodominant proteins, Gag, Env and Tax. Moreover, HBZ-specific humoral immune responses did not show any association with HTLV-1-related disease outcomes, while HTLV-1-infected AC and ATL patients from HAM/TSP patients could be discriminated based on the differential antibody responses for Gag, Env and Tax [31]. ATL patients showed lower mean immunoreactivity against HBZ compared to HAM/TSP patients (Figure 1A, Table 2). This is consistent with our previous report that ATL patients demonstrated lower levels of antibody responses to all three HTLV-1 immunodominant proteins, Gag, Env and Tax, compared to HAM/TSP patients [31]. This may be a consequence of a more global immunosuppressed state in ATL than HAM/TSP [35]. Interestingly, the distribution of an HBZ-specific antibody response by ATL subtype demonstrated that immunoreactivity against HBZ might differentiate the three clinical subtypes of ATL patients. Among ATL patients, there was a significant difference in the HBZ-specific antibody responses between patients with the chronic and lymphoma subtypes (Figure 1D). The median survival of patients with ATL is 20 weeks; patients with the acute subtype survive for a median of 13 weeks, patients with the lymphoma subtype survive for a median of 20 weeks, and patients with the chronic subtype survive for a median of 25 weeks [36]. The diagnostic criteria and clinical classification of the chronic ATL subtype includes more absolute lymphocytes with T lymphocytosis, and is associated with a better prognosis compared to the acute and lymphoma ATL subtypes [37]. Therefore, our results suggest that a subset of ATL patients generate HBZ-specific immune response (of low magnitude), which may delay disease progression. Since previous reports did not show any significant differences in antibody responses for HTLV-1 Gag, Env and Tax among ATL subtypes [31], it is important to validate these observations with HBZ in a larger sample of ATL patients that includes the less common subtype, smoldering ATL. In addition, since high expression of HBZ mRNA has been reported in ATL cells [19], it would be of interest to correlate anti-HBZ antibody responses with levels of HBZ mRNA. Unfortunately, PBMCs from ATL patients in this study were not available for analysis.

The lack of correlation of antibody responses for HBZ with proviral loads or HBZ mRNA expression might partially be the result of virological properties of HBZ itself. It has been reported that HBZ mRNA was detectable in PBMCs of HAM/TSP and ATL patients after culture, but mainly remains retained in the nucleus more than in the cytoplasm [38]. In primary ATL cell lines, only the spliced form of HBZ protein was detected in the nuclear fraction [39]. These reports suggest that persistence of HTLV-1 might be a consequence of reduced HBZ translation or limited localization of HBZ protein, and perhaps of reduced exposure of infected cells to HBZ-specific host immune responses. In other retrovirus infections such as human immunodeficiency virus (HIV), the regulatory protein Tat plays an important role in viral infectivity and pathogenicity [40], however Tat-specific antibody responses are detected only in a small number of HIV-infected individuals [41]. Similarly, antibody responses for HBZ were only observed in a subset of HTLV-1-infected individuals. Genetic factors, such as HLA, may also play a role in generation of a specific immune response, but larger numbers of HTLV-1-infected individuals with HBZ-specific humoral immune response will be required to further characterize the humoral immune response against HBZ in HTLV-1-associated diseases.

Antibody responses for HTLV-1 Gag, Env and Tax were elevated both in serum and CSF of HAM/TSP patients (Figure 3), consistent with previous reports [10,32]. By contrast, HBZ-specific antibody responses were only detected in CSF of HAM/TSP patients if there was a coincident serum antibody response against HBZ. These virus-specific antibodies in the CSF are either derived from the blood (leakage through the blood–brain-barrier) or alternatively, are synthesized locally within the CNS. Interestingly, the ratio of HBZ-specific antibody responses in CSF to serum was lower compared to ratios of antibody responses for HTLV-1 Gag, Env and Tax in CSF to serum (Figure 3). These results suggest that HBZ-specific antibody in the CSF is derived from the blood while antibodies for HTLV-1 Gag, Env and Tax are intrathecally synthesized. It has been previously reported that intrathecal antiviral antibody synthesis was confirmed by the presence of HTLV-1-specific antibodies and oligoclonal IgG in CSF [42-45] and that the lack of intrathecal antibody response to HTLV-1 in HAM/TSP correlates with higher proviral loads and worse outcome [46]. In the future it will be interesting to examine the relationship between CSF proviral load, and the presence or level of anti-HBZ antibody in CSF and to compare the clinical phenotype of HAM/TSP patients with and without CSF anti-HBZ antibodies.

Lastly, to further confirm the presence and the function of HBZ-specific antibody, we generated memory B cells producing HBZ-specific antibody from HAM/TSP patients with antibody response against HBZ. Since antigen-specific human memory B cells circulate at very low frequencies in peripheral blood, many researchers have relied on expansion and conversion of memory B cells into antibody-secreting cells by in vitro culture or development of alternative strategies [47-52]. In the present study, HBZ-specific antibody production was detected in memory B cells generated from all HAM/TSP patients who had antibody responses for HBZ in serum. As control, HBZ-specific antibody production was not detected in memory B cell cultures generated from HAM/TSP patients without HBZ-specific antibody response, while memory B cells generated from both HAM/TSP patients with and without HBZ-specific antibody response could produce antibodies specific to HTLV-I Gag, Env or Tax (data not shown). These results suggested that HTLV-1-specific memory B cells are maintained through an individual’s lifetime at levels that correlate with sustained serum antibody concentrations. Moreover, HBZ-specific antibody could significantly inhibit spontaneous lymphoproliferation of HAM/TSP patients without anti-HBZ response, including CD8^+^ T cell proliferation. HTLV-1 predominantly infects CD4^+^ T cells, but CD8^+^ T cells have also been shown to carry high proviral loads in HAM/TSP patients [53] and HBZ mRNA was detectable in both CD4^+^ and CD8^+^ T cells isolated from HAM/TSP patients (data not shown). It remains to be defined how HBZ-specific antibody inhibits CD8^+^ T cell proliferation in HAM/TSP patients. However, since humoral immunity is not limited to extracellular viral recognition but can neutralize a virus even within the cytosol of infected cells [54], HBZ-specific antibodies might therefore be able to interact with HBZ within HTLV-I infected cells. Further experiments including the nuclear retention and translation of HBZ mRNAs and the involvement of HBZ in lymphoproliferation of HAM/TSP patients would support these conclusions. Although we did not detect an inhibitory effect of HBZ-specific antibody on CD4^+^ T cell proliferation because this subset exhibits less spontaneous proliferation than CD8^+^ cells, the presence of antibody responses against HBZ was associated with less CD4^+^ T cell activation (frequency of CD25^+^ T cell subset) in HAM/TSP patients with anti-HBZ antibody responses. In CD8^+^ T cells, the frequency of CD25^+^ cells was also less in HAM/TSP patients with anti-HBZ antibody responses and approached statistical significance. These results demonstrated that HBZ-specific antibody responses have potentially beneficial roles in suppressing T cell activation in HAM/TSP patients. Since T cell activation is regulated through intricate molecular and immunological signaling networks, it will be of interest to determine how HBZ-specific antibody suppresses T cell activation of HAM/TSP patients.

## Conclusions

In summary, this is the first report demonstrating the presence of a humoral immune response to HBZ in the context of HTLV-1-infection. Characterization of the immune response against each of the HTLV-1 viral antigens will further improve our knowledge of virus-host interactions and the pathogenesis of HTLV-1-related disorders.

## Methods

### Subjects

The subjects for the present analysis were participants in research studies conducted at the National Institute of Neurological Disorders and Stroke (NINDS) at NIH, or the University of the West Indies (UWI), Kingston, Jamaica in collaboration with the National Cancer Institute (NCI), Bethesda, MD. Informed consent was written and obtained from each subject in accordance with the Declaration of Helsinki.

NIH subjects: Serum samples were obtained from a total of 92 subjects, including 25 HTLV-1-seronegative donors (NDs), 12 ACs and 55 HAM/TSP patients. CSF samples were obtained from 5 HAM/TSP patients. The study was reviewed and approved by the National Institute of Neurological Disorders and Stroke Institutional Review Board.

Jamaican subjects: All serum and plasma samples from study subjects were previously tested for HTLV by ELISA (Dupont, Wilmington DE) or EIA (Vironostika, Organo Teknika, Durham, NC); seropositive samples were previously tested by Western blot (Cambridge Biotech, Rockville MD or Genelabs Diagnostics HTLV-1 blot 2.4, Singapore). Serum/plasma samples were obtained from a total of 344 subjects, including 73 NDs, 133 HTLV-1 seropositive ACs, 89 ATL patients and 49 HAM/TSP patients. The NDs and ACs were selected from participants in a nested case–control study of risk factors for HTLV-1 seropositivity conducted among foodhandlers from Kingston and Clarendon parishes in 1987–1988 [55]. Samples from these subjects were obtained from either that study, or a previous seroprevalence study that these subjects participated in between 1985–1986 [56]. ATL subjects were selected from among ATL cases identified through an island-wide disease registry and referred to the UWI clinic in 1984–2006 [36]. For the current analysis, we randomly selected 30 subjects from each of the two ATL subtypes (acute and lymphoma) and 29 subjects from the ATL subtype (chronic) for a total of 89 subjects. In Jamaica, acute ATL is the most common subtype, accounting for 47% of ATL patients. The lymphoma and chronic subtypes occur in 27% and 21% of ATL patients, respectively. A fourth subtype, smoldering ATL, is uncommon, occurring in only 6% of Jamaican ATL patients (and were not included in this analysis) [36]. HAM/TSP subjects were selected among participants in an island-wide registry conducted in 1988–1998 [57]. The study protocols followed the human experimentation guidelines of the US Department of Health and Human Services and Institutional Review Board were approved by the NCI and UWI.

### LIPS assay

HTLV-1 cDNA clones for Gag, Env and Tax were inserted into pREN2, a mammalian *Renilla* luciferase expression vector, and generated as previously described [30]. A HTLV-1 cDNA clone for HBZ (AU1), which is a spliced form, was kindly gifted from Dr. Genoveffa Franchini (NCI, NIH, Bethesda, MD). Primers used for generation of HBZ/pREN2: HBZ-LIPS-F: 5′-gag gga tcc gcg gcc tca ggg ctg ttt cga t-3′; HBZ-LIPS-R: 5′-ctc tct aga tta ttg caa cca cat cgc ctc cag-3′. Each mammalian expression vector with the HTLV-1 gene was transfected into 293T cells using FuGENE®6 transfection reagent (Roche Diagnostics, Indianapolis, IN) [31]. The LIPS assay was performed as previously described [31]. Serum, plasma or CSF samples were diluted to 1:100. All anti-HBZ data from independent experiments were normalized using the LU values of positive control rabbit anti-HBZ serum. Cut-off values for anti-HBZ immunoreactivity were defined as an HBZ-LIPS antibody response that was in the 100 percentile of the values of the ND group (6853 LU). All anti-Gag, anti-Env and anti-Tax data from independent experiments were normalized using the LU values of positive control serum from a well-known HAM/TSP patient.

### HTLV-1 proviral DNA load

HTLV-1 proviral DNA load was measured using Viia™ 7 Real-Time PCR system (Applied Biosystems, Carlsbad, CA) as previously described [58]. DNA was extracted from PBMCs of HAM/TSP patients using QIAamp DNA Blood Mini Kit (QIAGEN, Germantown, MD), and 100ng of the sample DNA solution per well was analyzed by this system. All samples were performed in triplicate.

### HTLV-1 HBZ mRNA detection

Total RNAs were extracted from PBMCs of HAM/TSP patients by RNeasy® Mini Kit (QIAGEN), according to the manufacture’s instruction. 85 ng of total RNA was used to be converted into cDNA and amplified in a one step reaction using TaqMan® RNA-to-Ct™ 1-Step Kit (Applied Biosystems) according to the manufacturer’s instructions. The sequences of primers and probe for HBZ mRNA detection were as follows: (forward) 5′-aga acg cga ctc aac cgg-3′, (reverse) 5′-tga cac agg caa gca tcg a-3′ and (probe) 5′-tgg atg gcg gcc tca ggg ct-3′. As the probe for HBZ surrounded the splice junction site of HBZ mRNA, this method detected only the spliced form of HBZ. Hypoxanthine-guanine phosphoribosyltransferase (HPRT) was detected as an endogenous control. The HTLV-1-infected cell line MT-2 was used as a calibrator sample and the level of HBZ mRNA expression was then calculated using the comparative CT method on ViiA™ 7 software (Applied Biosystems).

### Generation of IgG^+^ memory B cells

IgG^+^ memory B cells were isolated from PBMCs using IgG^+^ memory B cells isolation kit (Miltenyi, Bergisch Gladbach, Germany). The memory B cells were seeded at 50 cells per wells in 96 U-bottom microplates in complete medium containing 2 ng of ODN 2006 (Invivogen, San Diego, CA) in the presence of EBV (30% supernatant of B95.8 cells) and irradiated allogeneic mononuclear cells (20,000 per well). After culture for two weeks, the production of HTLV-1-specific antibodies was screened in the culture supernatants of immortalized memory B cells using LIPS assay. The culture supernatants of memory B cells producing anti-HBZ were collected and stored at −807°C until use. After desalting, HBZ-specific IgG was isolated using HiTrap protein G columns (GE Healthcare, Uppsala, Sweden) and concentrated by Amicon® Ultra centrifugal filters (Millipore, Ireland).

### Western blot

The production of HBZ-specific antibody from memory B cell culture was further determined by western blot. The nuclear proteins were extracted from HTLV-1-infected (MT-2 and HUT102) and uninfected cell lines (Jurkat and MOLT-3), 293T cells and HBZ/pRen2-transfected 293T cells using Nuclear extract kit (Active Motif, Carlsbad, CA). Protein concentration was determined using Quick Start Bradford Protein Assay (BioRad, Hercules, CA). From each protein sample, 50 μg was electrophoresed through a NuPAGE® 4-12% Bis–Tris gel (Invitrogen). The gel was transferred to a nitrocellurose membrane (Invitrogen). After blocking with 3% BSA in TBS, the membrane was probed with B cell culture supernatant or rabbit anti-HBZ serum as positive control, and then probed with horseradish peroxidase-conjugated goat anti-human IgG (Santa Cruz Biotechnology, Santa Cruz, CA). The membrane was visualized by chemiluminescence using SuperSignal® West Pico Chemiluminescent substrate (Thermo Scientific, Rockford, IL) and analyzed the profile on Kodak digital science™ 1D image analysis software (Kodak, Rochester, NY). The intensity of HBZ proteins detected by B cell culture supernatant or rabbit anti-HBZ serum was normalized by the intensity of β-actin.

### Flow cytometry

For analysis of peripheral blood lymphocyte populations, patients’ PBMCs were stained with CD3, CD4, CD8 and CD25 (all from BD Biosciences, San Jose, CA). Flow cytometric analysis was performed using a LSR II (BD Biosciences). The data were analyzed using FlowJo software (Tree Star, San Carlos, CA).

### Lymphoproliferation assay

PBMCs were labeled with CFSE (CellTrace™ CFSE cell proliferation kit; Invitrogen) according to the manufacturer’s instruction, and plated at 2×10^5^ cells/well into 96 U-bottom microplates with 1 μg/ml of HBZ-specific IgG or human IgG as control. After culture for 6 days, the cells were stained with antibodies against CD3, CD4 and CD8 (all from BD Biosciences). The data were acquired on an LSRII flow cytometer (BD Biosciences).

### Statistical analysis

Race, gender and age information for each patient were organized into matrix form with the anti-HBZ data generated for each patient. Using Prism (GraphPad software), the Mann–Whitney Test was used to compare: the age distributions among the groups, anti-HBZ antibody levels between patient study groups (i.e., “ND”, “AC”, “ATL”, “HAM/TSP”), anti-HBZ antibody levels between ATL subtypes (i.e., “Acute”, “Chronic”, “Lymphoma”), anti-Gag, anti-Env and anti-Tax antibody levels between anti-HBZ positive and anti-HBZ negative groups, proviral DNA loads and HBZ mRNA expression between anti-HBZ positive and anti-HBZ negative groups, and frequencies of CD4^+^CD25^+^ and CD8^+^CD25^+^ T cells of NDs, HAM/TSP patients with and without antibody responses for HBZ. Still within Prism, the Chi-Square Test was used to compare the gender and racial distributions among the groups, numbers of anti-HBZ positive and anti-HBZ negative subjects between study groups and separately again to compare numbers of anti-HBZ positive and anti-HBZ negative patients between ATL subtypes. Again in Prism, Wilcoxson matched-pairs signed rank test was used to evaluate the inhibitory effects of HBZ-specific antibody on spontaneous lymphoproliferation in PBMCs of HAM/TSP patients. Lastly in Prism, Spearman’s correlation was used to evaluate both the association between anti-HBZ antibody levels and proviral DNA loads and the association between anti-HBZ antibody levels and HBZ mRNA expression. Using the statistical programming language “R” (http://www.r-project.org/), anti-HBZ data was log (base = 2) transformed then fit via the Four-Way Analysis of Variance (ANOVA) model with interactions; using race, gender, age and patient study group as the factors. Per race, patients were coded “Caucasian-descent”, “African-descent”, or “other”. Per gender, subjects were coded “male” or “female”. Per age, subjects were coded by quartiles as “young”, “young to middle-age”, “middle-age to senior”, or “senior”. Post-hoc testing was accomplished using the Tukey’s Honest Significant Difference method.

## Competing interests

The authors declare no competing financial interests. The views expressed are those of the authors and not necessarily those of the US Department of Health and Human Services, the NIH, or the FDA. Dr. Maloney’s contributions derived from her former affiliation with the National Cancer Institute, and not her current affiliation with the Food and Drug Administration.

## Authors’ contributions

YE-A performed most of the experimental work, statistical analysis and contributed to paper writing. AA performed the experimental work and contributed to paper writing. RM coordinated clinical work, analyzed gene expressions and contributed to discussion and paper writing. IB generated the HBZ/pRen2 plasmid and contributed to paper writing. KRJ performed statistical analysis and contributed to paper writing. PLG provided rabbit anti-HBZ sera and contributed to paper writing. EMM coordinated the identification of the subjects for this analysis and arranged for the selection of serum/plasma samples and contributed to discussion and paper writing. SJ supervised the project and contributed to discussion and writing. All authors read and approved the final manuscript.
